# Illness-related variables and abnormalities of resting-state brain activity in schizophrenia

**DOI:** 10.3389/fpsyt.2024.1458624

**Published:** 2024-08-06

**Authors:** Luigi Giuliani, Pasquale Pezzella, Giulia Maria Giordano, Leonardo Fazio, Armida Mucci, Andrea Perrottelli, Giuseppe Blasi, Mario Amore, Paola Rocca, Alessandro Rossi, Alessandro Bertolino, Silvana Galderisi, Mario Maj

**Affiliations:** ^1^ Department of Psychiatry, University of Campania “Luigi Vanvitelli”, Naples, Italy; ^2^ Department of Basic Medical Science, Neuroscience and Sense Organs, University of Bari ‘Aldo Moro’, Bari, Italy; ^3^ Department of Medicine and Surgery, Libera Università Mediterranea Giuseppe Degennaro, Casamassima, Italy; ^4^ Department of Neurosciences, Rehabilitation, Ophthalmology, Genetics and Maternal and Child Health, Section of Psychiatry, University of Genoa, Genoa, Italy; ^5^ Department of Neuroscience, Section of Psychiatry, University of Turin, Turin, Italy; ^6^ Department of Biotechnological and Applied Clinical Sciences, Section of Psychiatry, University of L’Aquila, L’Aquila, Italy

**Keywords:** cognitive impairments, negative symptoms, expressive deficit domain, motivational deficit domain, biomarkers, resting-state fMRI

## Abstract

**Background:**

The development of neuroimaging biomarkers in patients with schizophrenia (SCZ) requires a refined clinical characterization. A limitation of the neuroimaging literature is the partial uptake of progress in characterizing disease-related features, particularly negative symptoms (NS) and cognitive impairment (CI). In the present study, we assessed NS and CI using up-to-date instruments and investigated the associations of abnormalities in brain resting-state (rs)-activity with disease-related features.

**Methods:**

Sixty-two community-dwelling SCZ subjects participated in the study. Multiple regression analyses were performed with the rs-activity of nine regions of interest as dependent variables and disease-related features as explanatory variables.

**Results:**

Attention/vigilance deficits were negatively associated with dorsal anterior cingulate rs-activity and, together with depression, were positively associated with right dorsolateral prefrontal cortex rs-activity. These deficits and impairment of Reasoning/problem-solving, together with conceptual disorganization, were associated with right inferior parietal lobule and temporal parietal junction rs-activity. Independent of other features, the NS Expressive Deficit domain was associated with the left ventral caudate, while the Motivational Deficit was associated with the dorsal caudate rs-activity.

**Conclusion:**

Neurocognitive deficits and the two negative symptom domains are associated with different neural markers. Replications of these findings could foster the identification of clinically actionable biomarkers of poor functional outcomes.

## 1 Introduction

Schizophrenia is a complex and heterogeneous mental disorder in terms of pathophysiology, clinical presentation, and functional outcome (1–5). This disorder greatly impacts several aspects of functional outcome, such as social, vocational and independent living skills (6–14). Among illness-related aspects, negative symptoms and cognitive impairments, seem to represent the major predictors of a poor functional outcome, more than positive symptoms, disorganization and depression (7, 9, 12, 15–21). Negative symptoms are core features of schizophrenia, they often remain stable through the different phases of the illness largely contributing to the disability of the patients (15, 22–27). They cluster into two domains: the Motivational Deficit, which includes avolition, anhedonia and asociality, and the Expressive Deficit, which includes blunted affect and alogia (22, 24, 28–30). Although not part of the diagnostic criteria, neurocognitive dysfunctions are present in most subjects with schizophrenia (SCZ) and in their non-affected relatives and have a significant impact on daily functioning (31–35). Different neurocognitive domains, such as attention, speed of processing, working memory, visuospatial learning and memory, verbal learning and memory, reasoning and problem solving and executive functions, are impaired in patients with schizophrenia (36).

Several studies investigated brain alterations that might underlie different clinical features of schizophrenia. Functional magnetic resonance imaging (fMRI) during resting has been widely used to gather valuable information on the brain activity and connectivity when the brain does not perform any task (37–40).

Negative symptoms are linked to different alterations in brain activity and connectivity within several areas and circuits (28, 41–44). The Motivational Deficit domain seems to be related to brain alterations in pathways implied in different aspects of motivation, which are often impaired in subjects affected by schizophrenia. These pathways mainly involve brain areas within the “motivational value system or reward” and the “motivational salience” circuits (28)). In particular the Motivational Deficit domain has been found to be associated with dysfunctions of the resting-state functional connectivity within the right ventral putamen-medial orbitofrontal cortex pathway (45), the cingulo-opercular pathway (46), the left dorsal caudate-dorsolateral prefrontal cortex pathway (47), the precuneus (48), and the medial prefrontal and temporal pathways (49), as well as with with altered functional connectivity between the ventral tegmental area and the right ventro-lateral prefrontal cortex, the bilateral insular cortex, and the right lateral occipital complex (50).

The Expressive Deficit domain seems to be associated with alterations in neurodevelopmental processes (22, 51–53). Very few rs-fMRI studies investigated the neural correlates of the Expressive Deficit domain, showing that abnormalities in fronto-polar cortex functional connectivity could be associated with this domain (54, 55). Brain areas most probably involved in the pathophysiology of this domain are the cortical motor areas, the ventrolateral prefrontal cortex, the rostral anterior cingulate cortex, the amygdala, and the basal ganglia (41).

Neurocognitive impairments have been regarded as the result of the effect of different alterations in cortico–cerebellar–thalamic circuits involved in neurodevelopment, neuronal maturation and neuroplasticity (56–58). Recent meta-analyses reported that the impairment in neurocognitive performance in schizophrenia is correlated with the decreased resting-state activity of different neural networks, such as the Default Mode Network (DMN), the visual network, the salience network (including the left amygdala, left insula, bilateral inferior frontal gyrus, and right anterior cingulate cortex), and some other brain areas, such as the supplementary motor area and the putamen (59, 60). Most studies included in these meta-analyses measured cognitive functions by using assessment tools that do not separately and systematically evaluate all the cognitive domains found impaired in schizophrenia. Actually, different neurocognitive domains seem to be related with different brain alterations. For instance, working memory deficits seem to be associated with a functional dysconnectivity between the left and right Central Executive Networks, the visuospatial sketchpad (right ventro-lateral prefrontal cortex and intraparietal sulcus) and the phonologic Loop (left ventro-lateral prefrontal cortex and temporo-parietal junction) (61); deficits in speed of processing are associated with resting-state connectivity in the bilateral postcentral gyri, paracentral lobule (62), the right superior frontal gyrus and left postcentral gyrus (63).

Resting state fMRI abnormalities have also been investigated in relationship with other psychopathological dimensions of SCZ.

The severity of positive symptoms has been associated with an increased cerebral blood flow (CBF) in frontotemporal-parietal regions, posterior cingulate gyrus, lingual gyrus (64) and in subcortical regions such as the lenticular nucleus (i.e., pallidum and putamen), caudate, striatum, and hippocampus (65). These symptoms have also been associated with aberrant functional connectivity in the DMN, frontotemporal and auditory networks in SCZ (66).

Severity of disorganization in previous fMRI resting-state studies has been reported in association with the hyperactivity of the language network, i.e., the inferior frontal gyrus, superior temporal gyrus and inferior parietal lobe (67, 68). Auditory verbal hallucinations have also been associated with abnormalities in these areas (69, 70), suggesting a key role of the language network in the pathophysiology of positive symptoms and disorganization. Moreover, conceptual disorganization in schizophrenia has been reported to be strongly associated with a widespread brain dysconnectivity at rest (66, 71), involving the right lingual gyrus, left precuneus, left middle temporal gyrus, left posterior superior temporal sulcus, and right fusiform gyrus (67, 71–73).

To our knowledge, no study investigated the specific neural correlates of depressive symptoms in schizophrenia using rs-fMRI. Only one study using task-based fMRI showed a positive association of the activity of left thalamus, putamen, globus pallidus, insular lobe, inferior frontal gyrus, middle frontal gyrus and precentral gyrus, with depressive symptoms severity in schizophrenia (74).

Despite the large number of studies investigating the resting-state neural activity in schizophrenia, drawing clear conclusions about the neurobiological abnormalities associated to specific psychopathological dimensions of schizophrenia is still difficult. This could be mainly linked to: 1) the different conceptualization of the psychopathological dimensions across studies; 2) the presence of confounding factors, such as the impact of antipsychotic treatments on the neural activity of SCZ.

Our study aimed to improve the knowledge on the neural pathways underling the complex clinical presentation of schizophrenia investigating the relationships between illness-related features and abnormalities in rs brain activity. We hypothesized that deficits in different neurocognitive domains, evaluated through a comprehensive battery of standardized tests, and different aspects of psychopathology, characterized by using up-to-date instruments based on most recent conceptualizations, could identify distinct alterations of rs brain activity.

## 2 Methods

### 2.1 Participants

Subjects participating in the current study were from the same cohort as a previously published study conducted by Giordano et al. (37).

Sixty-six SCZ were enrolled across five Italian university psychiatric clinics that joined the Italian Network for Research on Psychoses (NIRP), as detailed in Giordano et al. (37).

The inclusion criterion was a diagnosis of schizophrenia according to DSM-IV, confirmed by the Structured Clinical Interview for DSM IV-Patient version (SCID-I-P). Exclusion criteria were: (a) a history of head injury resulting in loss of consciousness; (b) a history of moderate-to-severe intellectual disability or neurological diseases; (c) a history of alcohol and/or substance abuse in the previous six months; (d) current pregnancy or breastfeeding; (e) an inability to provide informed consent; and (f) treatment modifications and/or hospitalization due to symptom exacerbation in the previous three months.

All subjects were requested to provide a written informed consent to take part in the study after receiving a thorough description of the study’s procedures. These procedures were in line with the Helsinki Declaration of 1975, as updated in 2008, and to the ethical requirements of the relevant national and institutional committees on human experimentation. This study was approved by the Ethics Committee of the Università degli Studi della Campania “Luigi Vanvitelli”— Azienda Ospedaliera Universitaria “Luigi Vanvitelli”, A.O.R.N. “Ospedali dei Colli” and by the Ethics Committees of the involved collaborating institutions.

### 2.2 Psychopathological assessment

In the present study, the Positive and Negative Syndrome Scale (PANSS) (75) was used to assess positive, negative, and disorganization dimensions. In particular, the positive dimension was calculated according to Wallwork and colleagues (76) by adding up the scores of the items “delusions” (P1), “hallucinatory behavior” (P3), “grandiosity” (P5), and “unusual thought” (G9) and the disorganization dimension was assessed by the PANSS item “conceptual disorganization” (P2), in order to prevent overlap with cognitive impairment (7).

Negative symptoms were assessed using the Italian version of the Brief Negative Symptom Scale (BNSS) (77, 78). The BNSS is a scale developed according to the recent conceptualization of negative symptoms, in line with the NIMH-MATRICS Consensus Statement on Negative Symptoms (30). It explores all the domains of the negative construct, including avolition, anhedonia, asociality, blunted affect, and alogia, plus an additional aspect, “distress”, which evaluates the lack of normal experience of distressing and unpleasant emotions (30). The scale includes 13 items and 6 subscales (5 negative symptom subscales that include anhedonia, asociality, avolition, blunted affect, and alogia, and the control subscale that includes distress). The ratings for each item range from absent (0) to moderate (3) to extremely severe (6). In the present study, the “distress” subscale was subtracted from the overall score to calculate the negative symptom total score (78). The Motivational Deficit domain was obtained by adding the scores of the subscales of anhedonia, asociality, and avolition, and the Expressive Deficit domain was obtained by adding the scores of the alogia and blunted affect subscales.

We also used the Calgary Depression Scale for Schizophrenia (CDSS) to evaluate depression (79).

For all these evaluations, higher scores indicated more severe symptoms.

Neurocognitive functions were assessed using the Measurement and Treatment Research to Improve Cognition in Schizophrenia (MATRICS) Consensus Cognitive Battery (MCCB) (80, 81). This battery evaluates six neurocognitive domains: (a) processing speed (PS); (b) attention and vigilance (A/V); (c) working memory (WM); (d) verbal learning (SLe); (e) visual learning (VLe); (f) reasoning and problem solving (RPS). Raw scores on the MCCB were standardized to T-scores, corrected for age and gender, based on the Italian normative sample of community participants (82). For cognitive domains including more than one measure a summary score for the domain was calculated by summing the T-scores of the tests included in that domain and then standardizing the sum to a T-score. The same standardization procedure was adopted for the Neurocognitive Composite score. For all domains and composite scores, a T value of 50 is the normative mean and 10 is the SD. The lower the T value the more impaired the performance on the considered domain.

### 2.3 MRI data acquisition and pre-processing and ROI selection

Information regarding resting-state (rs) fMRI data acquisition and pre-processing, as well as regarding ROI selection procedure, are reported in the **Supplementary Materials**. In the present study we selected the ROIs whose rs activity differed between SCZ and healthy controls (HC) in the study by Giordano and colleagues (37). The ROIs were the following: dorsolateral prefrontal cortex (DLPFC), the inferior parietal lobule (IPL), the temporo-parietal junction (TPJ), the dorsal anterior cingulate cortex (dACC), the ventral caudate (vCa), and the dorsal caudate (dCa). Coordinates and size of these ROIs are summarized in **Supplementary Materials** (**Supplementary Table S1**).

### 2.4 Statistical analyses

Demographic continuous variables were reported as mean ± standard deviation (SD), while categorical variables were reported as frequencies.

We conducted separate stepwise multivariate regression analyses to explore potential illness-related variables with the highest associations with the rs-fMRI activity of those brain area that we previously found to differ between SZs and HCs. Specifically, we tested nine different regression models, one for each ROI that differed between SCZ and HCs (right IPL, right TPJ, right DLPFC, right and left dACC, right and left vCa, and right and left dCA). For each regression model, we used the rs activity of the ROI as dependent variables and PANSS positive dimension and disorganization, BNSS motivational deficit, BNSS expressive deficit, CDSS total score and the six MCCB neurocognitive domains (PS, A/V, WM, SLe, VLe and RPS) as independent variables. We could not include in the regression models the chlorpromazine equivalent dose, since we had this information only for a part of the participants. Therefore, to rule out the possible effects of pharmacological therapy on our results, we conducted Pearson’s correlations between chlorpromazine equivalent dose and the variables which had been included in the regression models.

The Statistical Package for the Social Sciences (IBM SPSS Statistics), Version 25, was used to conduct the statistical analyses.

## 3 Results

### 3.1 Sample characteristics

62 subjects with schizophrenia were included in the present analysis. The mean age of the sample was 37.92 ± 10.58 years. Participants had absent or mild positive symptoms and disorganization, and mild to moderate severe negative symptoms. Overall, patients showed deficits in all neurocognitive domains. Almost all patients were treated with antipsychotics, mostly second-generation drugs. **Table 1** shows the demographic and clinical characteristics of the study sample.

**Table 1:** Sociodemographic and clinical characteristics of patients with schizophrenia included in the study (N=62).

Age (mean ± SD)	37.92 ± 10.58
Education (mean ± SD)	12.56 ± 3.15
Gender (M/F)	37/25
PANSS Total score (mean ± SD)	60.20 ± 19.54
PANSS Positive (mean ± SD)	7.59 ± 3.64
PANSS Disorganization (P2 item) (mean ± SD)	1.84 ± 0.97
BNSS Total score (mean ± SD)	28.00 ± 17.61
BNSS Motivational Deficit (mean ± SD)	16.98 ± 9.77
BNSS Expressive Deficit (mean ± SD)	9.21 ± 7.94
CDSS total score (mean ± SD)	3.95 ± 3.98
MCCB – Speed of processing (mean ± SD)	29.77 ± 10.67
MCCB – Attention and vigilance (mean ± SD)	38.33 ± 11.70
MCCB – Working memory (mean ± SD)	33.93 ± 11.99
MCCB – Verbal learning (mean ± SD)	37.48 ± 10.77
MCCB – Visual learning (mean ± SD)	32.07 ± 14.87
MCCB – Reasoning and Problem Solving (mean ± SD)	36.07 ± 10.50
MCCB – Neurocognitive composite Score (mean ± SD)	28.63 ± 12.25
Type of AP medication (%)	77.4% second-generation AP; 10.5% first-generation AP; and 12.1% both
Chlorpromazine Equivalent daily dose	425.00 ± 233.68

PANSS, Positive and Negative Syndrome Scale; BNSS, The Brief Negative Symptom Scale; CDSS, The Calgary Depression Scale for Schizophrenia; MCCB, Measurement and Treatment Research to Improve Cognition in Schizophrenia (MATRICS) Consensus Cognitive Battery; AP, antipsychotics.

### 3.2 Regression analyses

We found that neurocognitive functions and negative symptom domains were associated with alterations of the rs-fMRI activity in several brain regions.

A reduced rs-fMRI activity in the bilateral dACC was associated with a worse performance in A/V domain (right dACC: β = 0.298, p = 0.036; left dACC: β = 0.295, p = 0.038), independently from psychopathology and deficits in other cognitive domains. Furthermore, a reduced rs-fMRI activity in the right DLPFC was associated with a worse performance in the A/V domain (β =0.279, p =0.037) and a reduced severity of depressive symptoms (β = 0.367, p = 0.009). An increased rs-fMRI activity in the right IPL and TPJ was associated with a worse performance in A/V (IPL: β = -0.416, p = 0.013; TPJ: β = -0.382, p = 0.021), better performance in reasoning and problem-solving (IPL: β = 0.549, p = 0.001; TPJ: β = 0.497, p = 0.003), and a reduced severity of the conceptual disorganization (IPL: β = -0.303, p = 0.023; TPJ: β = -0.382, p = 0.005). As to the relationships between rs-fMRI activity and negative symptom domains, a reduced rs-fMRI activity in the left vCA was associated with a higher severity of the Expressive Deficit (β = -0.398, p = 0.004); a reduced ars-fMRI activity of the bilateral dCA was associated with a higher severity of the Motivational Deficit (right: β = -0.341, p = 0.015; left: β = -0.347, p = 0.014).

The results of regression analyses are reported in **Table 2**.

**Table 2:** Results of stepwise regression analyses.

					t	p
		B	SE	Beta		
**R daCC**	A/V	.101	.047	.298	2.162	.036
**L daCC**	A/V	.109	.051	.295	2.139	.038
**R DLPFC**	CDSS total	.364	.133	.367	2.734	.009
	A/V	.097	.045	.279	2.152	.037
**R IPL**	RPS	.120	.034	.549	3.481	.001
	A/V	-.084	.033	-.416	-2.586	.013
	PANSS - p2	-.714	.305	-.303	-2.343	.023
**R TPJ**	PANSS - p2	-1.047	.351	-.382	-2.986	.005
	RPS	.126	.040	.497	3.188	.003
	A/V	-.090	.038	-.382	-2.400	.021
**R vCA**	–	–	–	–	–	–
**L vCA**	BNSS - Expressive Deficit	-.203	.068	-.398	-3.002	.004
**R dCA**	BNSS - Motivational Deficit	-.095	.038	-.341	-2.516	.015
**L dCA**	BNSS - Motivational Deficit	-.091	.036	-.347	-2.565	.014

R, right; L, left; DLPFC, dorsolateral prefrontal cortex; IPL, inferior parietal lobule; TPJ, temporoparietal junction; daCC, dorsal anterior cingulate cortex; vCa, ventral caudate; dCa, dorsal caudate; A/V, attention/vigilance; RPS, reasoning and problem solving; PANSS, Positive and Negative Syndrome Scale.

### 3.3 Control analysis

The chlorpromazine equivalent dose was available only for 50 subjects (81% of the sample). We found that the chlorpromazine equivalent dose did not correlate with any of the variables included in the regression models (**Table 3**).

**Table 3:** Control analyses (N=50).

	Equivalent of chlorpromazine	
	Pearson’s r	p
**PANSS p2**	.214	.148
**Motivational Deficit**	.033	.832
**Expressive Deficit**	.004	.982
**CDSS total**	.248	.093
**A/V**	.184	.206
**RPS**	.120	.407
**R DLPFC**	.262	.066
**R IPL**	-.248	.082
**R TPJ**	-.138	.341
**R daCC**	.182	.206
**L daCC**	.150	.297
**R vCA**	-.080	.582
**L vCA**	-.117	.419
**R dCA**	.152	.292
**L dCA**	.205	.153

PANSS, Positive and Negative Syndrome Scale; A/V, attention/vigilance; RPS, reasoning and problem solving; R, right; L, left; DLPFC, dorsolateral prefrontal cortex; IPL, inferior parietal lobule; TPJ, temporoparietal junction; daCC, dorsal anterior cingulate cortex; vCa, ventral caudate; dCa, dorsal caudate.

## 4 Discussion

The current study aimed to investigate the relationships between the illness-related features of schizophrenia and abnormalities in resting-state brain activity.

The results of our study highlighted that positive symptoms were not associated with the rs brain activity in the selected ROIs, while cognitive impairment and negative symptoms had the highest number of associations. The lack of associations between positive symptoms and rs brain activity should be interpreted in the light of the characterization of our sample which is mainly composed by chronic patients, with absent or mild positive symptoms, but moderate to severe negative symptoms and cognitive deficits.

Our results concerning the associations of negative symptoms and cognitive deficits with alterations of rs-fMRI activity in different ROIs are in line with recent literature reporting that, although these two aspects are often associated in chronic patients, they represent separate constructs subtended by alterations of different neural pathways (83).

Among neurocognitive deficits, impairment in the A/V domain was related to the reduced rs-activity of the right DLPFC and bilateral daCC, and the increased activity of the right TPJ and IPL. The direct relationship between A/V and the activity of the right DLPFC and bilateral daCC is in line with the majority of previous literature results (84–87), although a few studies did not report this association (88, 89). Moreover, the DLPFC and the daCC are strongly related to each other, forming nodes in several networks, and they are involved in different cognitive processes, particularly in the A/V (84, 87, 90–93). While the activity of the bilateral daCC was predicted only by cognitive dysfunctions, the reduced activity of the right DLPFC was associated also with a lower severity of depressive symptoms. This result could be interpreted in the light of a previous study that investigated the specific neural correlates of depressive symptoms in schizophrenia using a task-based fMRI (74). This study showed that the activation of the middle frontal gyrus (which roughly corresponds to the DLPFC), as well as the inferior and precentral gyri, the left thalamus, putamen, globus pallidus and insular lobe in response to fearful expressions during an implicit affect processing task (74) was associated with the severity of depressive symptoms. However, drawing conclusion on the relationship between depressive symptoms in schizophrenia and DLPFC is difficult since the literature on the topic is scarce (74). Therefore, further studies are needed, in order to replicate these findings.

Furthermore, deficits in the A/V, together with deficits in RPS, and conceptual disorganization were associated with the rs activity of the right TPJ and the right IPL. The inverse relationship between the activity of these regions and attention in SCZs is in line with previous findings showing that, in these patients, lower levels of attention were associated to increased activity of the right TPJ and right IPL, and to a reduced connectivity between the right TPJ and other brain regions such as posterior cingulate cortex or DLPFC (94–97). Evidence from neuroimaging studies suggested that the right TPJ and IPL are connected to each other and represent nodes in several brain networks, such as default mode network and the ventral attention network (98). This pathway is involved in a broad range of functions, such as the maintaining and reorienting of attention, reasoning and problem solving, memory, executive functions and social cognition (98–105). Moreover, the activity of IPL and TPJ is strongly lateralized (98). Indeed, in line with our findings, the literature suggests that the A/V functions are sustained by the right lateralized activity of the IPL and TPJ, while inconsistent findings are reported on the RPS functions, which are reported either to be subtended by lateralized left brain activity (106, 107), either not-lateralized brain activity (108–111). Furthermore, according to previous studies (67, 112, 113), we found that the rs hyperactivity in the right IPL and TPJ was associated also with a better performance in RPS and less severe conceptual disorganization. This finding might suggest a partial overlap between disorganization and some cognitive functions, such as A/V and RPS, as reported in some studies (114–119).

In our study the severity of negative symptoms was related to the rs hypoactivity of the caudate. In particular, the Expressive Deficit was associated to the activity of the right ventral caudate, while the Motivational Deficit to the bilateral dorsal caudate. This finding, in line with the literature, supports the hypothesis that the two negative symptom domains might show different neurophysiological correlates (22, 37, 50, 51, 120, 121). Particularly, the relationship between the severity of the Expressive Deficit domain and the resting-state hypoactivity of the left vCa in SCZs was already showed in our previous study (37), but it is in contrast with the literature on topic, that reported associations between the ventral caudate hypoactivity and the Motivational Deficit domain or the negative symptoms belonging to it (42, 122–133). To our knowledge only one rs-fMRI study already reported an association between alterations of the caudate activity and the severity of Expressive Deficit (134), without differentiating the dorsal and the ventral part of the nucleus. On the other side, we found an inverse association between the severity of Motivational Deficit domain and the rs activity of bilateral dorsal caudate. Our findings confirm the results of previous studies which reported the same correlation pattern in SCZs (37, 43, 135), thus supporting the hypothesis of dysfunctions within the motivational patwhay. Indeed, the dorsal caudate is part of the dorsal striatum and it is involved in the motivational value system. Furthermore, this brain region is engaged in coding associations between actions/stimuli and outcomes in goal-directed behaviors and in selecting actions based on their currently predicted reward value (136).

It is important to acknowledge some limitations that may affect the generalizability and reproducibility of the study findings. First, the sample size was relatively small, and included only clinically stable, treated subjects, thus limiting the possibility of generalizing the results. Therefore, further studies with larger samples, including drug-naïve subjects, are needed to replicate these findings. In addition, although we performed control analyses investigating the effect of chlorpromazine equivalent dose on the activity of the ROIs and the clinical features involved in the analyses, we could not include this variable into the regression models since we had this information only for a part of the participants. Therefore, additional studies including drug-naïve subjects are needed to confirm our findings. Another limitation is the reliance solely on cross-sectional data. Longitudinal designs offer advantages in enhancing the power of associations between brain measures and clinical and cognitive variables, as they allow for the observation of changes over time within the same individuals. Future research could benefit from incorporating additional timepoints per subject to strengthen the observed relationships (137). Additionally, our study was limited to resting-state fMRI data. Resting-state fMRI could overcome issues related to the study of task-related activation/functional connectivity that might result in specious findings due to the poor intellectual capacities or memory impairments frequently present in subjects with schizophrenia. However, task-based fMRI has been shown to yield stronger predictions of behavior, particularly cognition, and incorporating such paradigms in future research may provide a more comprehensive understanding of the neural correlates of negative and cognitive symptoms (138). Therefore, future studies should consider using both resting-state and task-based fMRI to improve the robustness and generalizability of the findings.

## 5 Conclusions

In conclusion, the results of the present study highlight that a detailed and comprehensive assessment of psychopathology and cognitive performance is a crucial step to improve our knowledge about neural correlate of schizophrenia.

## Data availability statement

The raw data supporting the conclusions of this article will be made available by the authors, without undue reservation.

## Ethics statement

The studies involving humans were approved by Ethics Committee of the Università degli Studi della Campania”Luigi Vanvitelli”—Azienda Ospedaliera Universitaria “Luigi Vanvitelli”, A.O.R.N. “Ospedali dei Colli” (protocol code 202, 10 March 2020). The studies were conducted in accordance with the local legislation and institutional requirements. Written informed consent for participation in this study was provided by the participants’ legal guardians/next of kin.

## Author contributions

LG: Conceptualization, Data curation, Formal analysis, Investigation, Methodology, Writing – original draft, Writing – review & editing. PP: Conceptualization, Data curation, Formal analysis, Investigation, Methodology, Writing – original draft, Writing – review & editing. GG: Conceptualization, Data curation, Formal analysis, Investigation, Methodology, Writing – original draft, Writing – review & editing. LF: Conceptualization, Data curation, Formal analysis, Investigation, Methodology, Writing – original draft, Writing – review & editing. AM: Conceptualization, Investigation, Writing – original draft, Writing – review & editing. AP: Conceptualization, Investigation, Writing – original draft, Writing – review & editing. GB: Conceptualization, Investigation, Writing – original draft, Writing – review & editing. MA: Conceptualization, Investigation, Writing – original draft, Writing – review & editing. PR: Conceptualization, Investigation, Writing – original draft, Writing – review & editing. AR: Conceptualization, Investigation, Writing – original draft, Writing – review & editing. AB: Conceptualization, Investigation, Writing – original draft, Writing – review & editing. SG: Conceptualization, Investigation, Writing – original draft, Writing – review & editing. MM: Conceptualization, Investigation, Writing – original draft, Writing – review & editing.

## Group members of Italian Network for Research on Psychoses

Antonio Melillo (University of Campania “Luigi Vanvitelli”, Naples), Cecilia Riccardi, Cristiana Montemagni, Daria Pietrafesa, Edoardo Caporusso, Eleonora Merlotti, Elisa Del Favero (University of Turin), Francesca Pacitti, Francesco Brando, Giuseppe Piegari, Marco Papalino, Martino Belvedere Murri, Noemi Sansone, Paola Bucci, Pietro Calcagno, Raffaella Romano (University of Bari), Rodolfo Rossi, Simone Cattedra (University of Genoa), Valentina Socci (University of L’Aquila), Vitalba Calia.
