# Supplementary material for: Illness-related variables and abnormalities of resting-state brain activity in schizophrenia
Source: Front Psychiatry. 2024 Aug 6;15:1458624. doi: 10.3389/fpsyt.2024.1458624 (PMC11333936; doi:10.3389/fpsyt.2024.1458624)
Supplement: Supplementary file 1 [file Table_1.docx]

**Supplementary data**

Methods - MRI Data Acquisition and Pre-Processing Pag. 2

Methods - ROIs selection procedure Pag. 3

Table S1. Regions of Interest description Pag. 4

**MRI Data Acquisition and Pre-Processing**

MRI evaluations were performed at five different sites and with six different 3 Tesla scanners. For all participants, we collected one sMRI and a resting-state-based functional MRI (rs-fMRI). For the sMRI, the T1-weighted structural images used the SPGR or MPRAGE sequences. Gradient-echo echo-planar imaging sequence was used to acquire images during the rs-fMRI acquisition (300 s, 150 volumes). For the sMRI, data processing was performed using the Computational Anatomy Toolbox 12 (CAT12, Structural Brain Mapping group, Jena University Hospital, Jena, Germany - http://www.neuro.uni-jena.de/cat12/, accessed on 20 May 2022) included in SPM12 (Statistical Parametric Mapping, Institute of Neurology, London, UK - https://www.fil.ion.ucl.ac.uk/spm/software/spm12/, accessed on 20 May 2022). The T1-weighted images were normalized on a standard brain (MNI152) using a diffeomorphic registration algorithm (DARTEL) and segmented into different tissue classes (gray matter, white matter, and cerebrospinal fluid) based on probability maps. All images were then modulated through Jacobian determinants to preserve initial volumes and smoothed with a 3 mm isotropic Gaussian filter. For the purpose of analysis, we used segmented gray matter images from sMRI, which reflect the gray matter volume (GMV) information of the whole brain. The quality-based inclusion criteria were as follows: an absence in the raw images of technical artifacts, such as blurring, ringing, wrapping, and incomplete head coverage, and an absence in the segmented images of excessive noise, poor image contrast, and/or inadequate boundaries.

To compensate for differences between the scanners in the MRI acquisition window, individual gray matter images were combined using the ImCalc toolbox in SPM12 with a multiplicative function in order to obtain a binary mask of voxels acquired only in each individual scanner. This mask containing only voxels common to all acquisitions (approximately 359,000 isotropic 1 mm voxels) was applied to all individual images. The resulting gray matter volume (GMV) maps were included in the multimodal group analyses. Individual total intracranial volume (TIV) was also calculated and used as a disturbance covariate in subsequent analyses.

The rs-fMRI data were preprocessed with SPM12. For each participant, functional volumes were realigned to correct for head movement. Individual motion parameters were extracted and used to calculate Friston 24 motion parameters. The realigned images were rescaled, co-registered to T1-weighted structural images, spatially normalized to a standard space (MNI 152) and masked using the gray matter mask. Finally, noise covariates, including Friston 24 head motion parameters, white matter signals, and cerebrospinal signals, were regressed and the images were smoothed with an isotropic 6 mm FWHM kernel. Wavelet despiking was performed to remove motion-related distortions. The quality-based inclusion criteria were as follows: an absence of scan artifacts and low head motion (translation > 3 mm, rotation > 3°, change in Framewise Displacement between volumes-FD > 0.05). The individual mean value of FD was calculated and used as a disturbance covariate in subsequent analyses.

**ROIs selection procedure**

For the extraction of signal time courses in each anatomical district, we used the Human Brainnetome Atlas (BNA) (100). The BNA atlas divided the brain into 246 regions of interest (ROIs), with 123 for each hemisphere, comprising 210 cortical and 36 subcortical ROIs. For each subject, we extracted the time courses from each of the 246 ROIs using the Data Processing Assistant for Resting-State fMRI (DPARSF) (http://www.rfmri.org (accessed on 20 May 2022)). The extracted data were then normalized within each subject by T-score transformation in order to minimize the global signal differences between subjects.

Starting from the 246 ROIs that emerged from the analysis, we selected 17 ROIs for each brain hemisphere, using an average value of the resting-state BOLD signal of areas belonging to the same brain region. The ROIs were the dorsolateral prefrontal cortex (DLPFC), the ventrolateral prefrontal cortex (VLPFC), the orbitofrontal cortex (OFC), the precuneus (PCun), the inferior parietal lobule (IPL), the temporo-parietal junction (TPJ), the superior temporal gyrus (STG), the ventral anterior insula (vaIC), the dorsal anterior insula (daIC), the posterior insula (pIC), the lateral occipital cortex (LOC), the dorsal anterior cingulate cortex (dACC), the amygdala (Amy), the nucleus accumbens (NaC), the ventral caudate (vCa), the dorsal caudate (dCa), and the putamen (Pu). In the present study we selected the ROIs whose resting-state activity differed between subjects with schizophrenia and healthy controls in the previous published work by Giordano et al 2023 (Giordano et al. 2023). The ROIs were the following: DLPFC, IPL TPJ, dACC, vCA, dCA. Coordinates and size of these ROIs are summarized in Table S1.

**Table S1. Regions of Interest description.**

| **Regions of Interest** | **Anatomical and modified**  **Cyto-architectonic descriptions** | **MNI(X,Y,Z)** |
| --- | --- | --- |
| **R DLPFC** | *A8dl, dorsolateral area 8* | [22,26,51] |
|  | *A9l, lateral area 9* | [13,48,40] |
|  | *A9/46d, dorsal area 9/46* | [30,37,36] |
|  | *A46, area 46* | [28,55,17] |
|  | *A8vl, ventrolateral area 8* | [42,27,39] |
| **R IPL** | *A39c, caudal area 39(PGp)* | [45,-71,20] |
|  | *A40rd, rostrodorsal area 40(PFt)* | [47,-35,45] |
|  | *A40c, caudal area 40(PFm)* | [57,-44,38] |
|  | *A40rv, rostroventral area 40(PFop)* | [55,-26,26] |
| **R TPJ** | *A39rv, rostroventral area 39(PGa)* | [53,-54,25] |
|  | *A39rd, rostrodorsal area 39(Hip3)* | [39,-65,44] |
| **R dACC** | *A32p, pregenual area 32* | [5,28,27] |
|  | *A24cd, caudodorsal area 24* | [4,6,38] |
|  | *A32sg, subgenual area 32* | [5,41,6] |
|  | *A32p, pregenual area 32* | [-6,34,21] |
|  | *A24cd, caudodorsal area 24* | [-5,7,37] |
|  | *A32sg, subgenual area 32* | [-4,39,-2] |
| **R vCa** | *vCa, ventral caudate* | [15,14,-2] |
| **L vCA** | *vCa, ventral caudate* | [-12,14,0] |
| **R dCa** | *dCa, dorsal caudate* | [14,5,14] |
| **L dCa** | *dCa, dorsal caudate* | [-14,2,16] |

*R = right; L = left; DLPFC = dorso-lateral prefrontal cortex; IPL = Inferior Parietal Lobule; TPJ = Temporo-Parietal Junction; dACC = dorsal anterior cingulate cortex; vCa = ventral caudate; dCa = dorsal caudate*

**References**

Giordano, G. M., P. Pezzella, L. Giuliani, L. Fazio, A. Mucci, A. Perrottelli, G. Blasi, M. Amore, P. Rocca, A. Rossi, A. Bertolino, S. Galderisi and P. Italian Network For Research On, 2023. "Resting-State Brain Activity Dysfunctions in Schizophrenia and Their Associations with Negative Symptom Domains: An fMRI Study." Brain Sci. 13. ^10.3390/brainsci13010083
